# Detection of *Helicobacter pylori* in City Water, Dental Units' Water, and Bottled Mineral Water in Isfahan, Iran

**DOI:** 10.1155/2013/280510

**Published:** 2013-03-31

**Authors:** Ahmad Reza Bahrami, Ebrahim Rahimi, Hajieh Ghasemian Safaei

**Affiliations:** ^1^Faculty of Veterinary Medicine, Islamic Azad University, Shahrekord Branch, Shahrekord, Iran; ^2^Department of Food Hygiene, Faculty of Veterinary Medicine, Islamic Azad University, Shahrekord Branch, Shahrekord, Iran; ^3^Department of Microbiology, Faculty of Medical Sciences, Isfahan University of Medical Sciences, Isfahan, Iran

## Abstract

*Helicobacter pylori* infection in human is one of the most common infections worldwide. However, the origin and transmission of this bacterium has not been clearly explained. One of the suggested theories is transmission via water. This study was conducted to determine the prevalence rate of *H. pylori* in tap water, dental units' water, and bottled mineral water in Iran. In the present study, totally 200 water samples were collected in Isfahan province and tested for *H. pylori* by cultural method and polymerase chain reaction (PCR) by the detection of the *ureC (glmM)* gene. Using cultural method totally 5 cultures were positive. Two out of 50 tap water samples (4%), 2 out of 35 dental units' water (5.8%) samples, and 1 out of 40 (2.5% ) from water cooler in public places were found to be contaminated with *H. pylori*. *H. pylori ureC* gene was detected in 14 (7%) of water samples including 5 tap water (10%), 4 dental units' water (11.4%), 1 refrigerated water with filtration, and 4 (10%) water cooler in public places samples. This may be due to the coccoid form of bacteria which is detected by PCR method.

## 1. Introduction

*Helicobacter pylori* is a gram-negative microaerophilic rod found in the human gastric mucosa and is associated with different digestive diseases, such as peptic ulcer, gastritis, and mucosa-associated lymphoid tissue lymphoma [1], and it is considered a risk factor in the development of gastric cancer [2].

*H. pylori* infection is frequently acquired during childhood, and symptoms such as vomiting and epigastric or recurrent abdominal pain are associated with *H. pylori* infection [3]. In developing countries, it is estimated that 70–90% of the population carries *H. pylori*, contrasting to 25–50% of infection among the inhabitants of developed countries [4]. It has been demonstrated that people living in developing countries acquire the infection earlier in life, when compared with individuals of the same age group in developed countries [5].

Despite the high incidence of the infection, the reservoirs and the transmission pathways of *H. pylori* to humans are still unclear, although multiple routes of transmission have been suggested [6]. The current literature suggests that the transmission of *H. pylori* occurs by person to person both via the oral-oral and fecal-oral routes [7]. Furthermore, many authors suggested that the human infection may occur by contaminated foods [8, 9]. Indeed, *H. pylori* has been detected from drinking water [10–13], sea water [14], and foods of animal origin, such as sheep and cow milk [15–17]. Epidemiological studies have shown that infection with *H. pylori* is associated with the level of sanitation, particularly water sanitation.

This study was conducted to determine the occurrence of *H. pylori* in tap water, dental units' water, water cooler in public places, refrigerated water with filtration, and bottled mineral water in Isfahan provinces, by means of a conventional bacteriological procedures and polymerase chain reaction (PCR).

## 2. Materials and Methods

### 2.1. Sample Collection

Isfahan province—with a population of 4,800,000 and area of 291,107,044 square kilometers—is the second biggest province of Iran and is located in the central part of Iran among Iran's central mountains eastern hillside of Zagros at the margin of the Zayande-Rood River. The drinking water of this province is supplied from the Zayande-Rood River which is considered to be surface water. It is probable that this water is contaminated with industrial and urban sewerage at the margin of this river. Other than refinement, water receives no treatment such as radiation. Taking all this, and given that this river is the only water source for companies producing bottled mineral water in this province, it is likely that one of the sources of microbial contamination in this area is water. In this study, a total of 200 samples including 50 samples of tape water, 35 samples of dental units, 30 samples of home refrigerator with filtration system, 25 samples tape water equipped with filtration system, 40 samples from water cooler in public places, and 20 samples of mineral bottled water were examined over a period of 6 months, from July to December 2011 from four different geographical regions of Isfahan province. For each region, 10–15 samples were collected in 1,000 mL glass bottles containing 0.5 g of sodium thiosulphate for dechlorination of the water. The 20 bottled mineral water samples were purchased from five different companies (using the same water system) on the day that the experiment was conducted.

### 2.2. Isolation of *Helicobacter pylori*

Samples of 1000 mL water collected in sterile glass flasks and transferred to laboratory within 2 hours. Samples were filtered through 0.045 *μ*m filter membrane (Albet Co.). Each membrane was then immersed into 2 mL of tryptic soy broth (TSB) for 1 h. After that each 2 mL TSB was taken and cultured for *H. pylori* and DNA extraction. Samples were cultured on Brucella agar (Merck, Germany) containing campylobacter selective supplement (5 mg/L, Merck), trimethoprim (0.25 mg/L), amphotericin B, sheep blood (5%), and 7% fetal calf serum (Sigma). After 72 h incubation at 37°C in microaerophilic condition (5% O_2_, 85% N_2_, 10% CO_2_) using MART system (Anoxamat, Lichtenvoorde, The Netherlands), the bacterial growth was tested and confirmed as *H. pylori* by gram staining, urease, and oxidase tests [1]. The isolates were identified as *H. pylori* were also positive, using the PCR assay. For comparison, a reference strain of *H. pylori* (ATCC 43504) was employed.

### 2.3. Detection of *Helicobacter pylori* Using PCR Method

DNA was extracted by a DNA isolation kit from mentioned TSB (Roche Applied Science, Germany) according to the manufacturer's instructions, and its density was assessed by optic densitometry. Extracted genomic DNA was amplified for the *ureC* (*glmM*) gene and detected with the specific primers HP-F: 5′-GAATAAGCTTTTAGGGGTGTTAGGGG-3′ and HP-R: 5′-AAGCTTACTTTCTAACACTAACGCGC-3′. The gene product was 294 bp. PCR reactions were performed in a final volume of 50 *μ*L containing 5 *μ*L 10 × buffer + MgCl_2_, 2 mM dNTP, 2 unit Taq DNA polymerase, 100 ng genomic DNA as a template, and 25 picomoles of each primer. PCR was performed using a thermal cycler (Eppendorf Co., Germany) under the following conditions: an initial denaturation for 10 minutes at 94°C; and 35 cycles for 1 minute at 94°C, 1 minute at 55°C, 1 minute at 72°C, and a final extension at 72°C for 10 minutes. The PCR products were electrophoresed through a 1.5% agarose gels (Fermentas, Germany) containing Ethidium bromide. A DNA ladder (Fermentas Co., Germany) was used to detect the molecular weight of observed bands under a UV lamp. All tests were performed in triplicate. Samples inoculated with *H. pylori* were used as positive controls.

### 2.4. Statistical Analysis

Data were transferred to Microsoft Excel spreadsheet (Microsoft Corp., Redmond, WA, USA) for analysis. Using SPSS 16.0 statistical software (SPSS Inc., Chicago, IL, USA), Chi-square test and Fisher's exact two-tailed test analysis was performed, and differences were considered significant at values of *P* < 0.05.

## 3. Results and Discussion

Using traditional bacteriologic methods, totally 5 cultures were positive. Two of 50 tap water samples (4%), 2 out of 35 dental units' water (5.8%) samples, and 1 of 40 (2.5%) from water cooler in public places were found to be contaminated with *H. pylori*. *H. pylori ureC* gene was detected in 14 (7%) of water samples including 5 tap water (10%), 4 dental units' water (11.4%), and 4 (10%) water cooler in public places samples (Table 1). Statistically significant differences (*P* > 0.05) were not observed in the prevalence of *H. pylori ureC* gene in water samples collected from different geographical regions of Isfahan province.

The association of serum antibodies against *H. pylori* with serum antibodies against two known waterborne pathogens hepatitis A virus [18] and Giardia [19] suggests that the infection may be waterborne or related to poor sanitary practices [20]. Klein et al. [21] studied the prevalence of *H. pylori* infection in 407 children (two months to 12 years old), in Lima, Peru. *H. pylori* infection rate was 56% among children from low-income families and 32% among those from high-income families. However, children from high-income families whose homes were supplied with municipal water were 12 times more likely to be infected than those from the same socioeconomic status whose water supply came from community wells. These results showed that the acquisition of *H. pylori* infection by Peruvian children was correlated with socioeconomic status, but additionally the municipal water supply seemed to be involved in the spread of infection among them. Indirect evidence that the transmission of *H. pylori* is waterborne is based upon four sets of data: (i) presence of DNA in water samples, (ii) observation of coccoid forms in water samples, (iii) survival of *H. pylori* in artificially contaminated water, and (iv) growth of *H. pylori* from water samples [20]. 

In the present study, only two tap water samples (4%) were found to be contaminated with *H. pylori* using traditional bacteriologic methods. *H. pylori* has rarely been isolated from water samples [22, 23]. In several studies no *H. pylori* was found in water samples [8, 24, 25]. This could be attributed to the fact that *H. pylori* can survive for short period of time in water [8, 20]. Moreover, the method employed for *H. pylori* isolation may lack sufficient sensitivity to recover very low numbers of *H. pylori* [2, 23, 26, 27]. 

Two out of 35 dental units' water samples were found to be contaminated with *H. pylori* using traditional bacteriologic methods. The presence of *H. pylori* associated with biofilms from wells, rivers, and water distribution systems has been reported by different investigators [28–32]. Biofilms are slimy films of bacteria, other microbes, and organic materials that cover underwater surfaces, particularly inside plumbing. This makes them rather inaccessible and provides a matrix difficult to be reached by disinfectants. The detachment of biofilms is the principal form of contamination of treated water [33, 34]. Taken together, these results suggest that biofilms in water distribution systems are responsible for the contamination of water.

In this study, *ureC *gene of *H. pylori* was detected in tap water, dental units' water, refrigerated water with filtration, and public cooler water samples. *H. pylori* DNA has been identified in several water sources using diverse gene targets. Drinking, river, sea, ground, and wastewater have provided positive results by PCR analysis [9, 14, 23, 24, 35–37]. The *H. pylori* DNA present in water samples could be from dead *H. pylori* cells or from VBNC forms, since culture is usually not possible. Water spiked with viable *H. pylori* cells rapidly led to the observation of coccoid forms [23, 27, 38, 39]. Whether the coccoid form of *H. pylori* is viable in the dormant state or is degenerative and undergoing apoptosis is still an unanswered question. Coccoid *H. pylori* appears to conserve the capacity to produce proteins for at least 100 days when stored at 4°C, in either phosphate-buffered saline (PBS) or distilled water [40]. It has been suggested that although the virulence of coccoid *H. pylori* induced by water decreases, the coccoid forms still retain a considerable urease activity and preserve adhering ability to epithelial cells. These coccoid forms induced by water have been capable of colonizing the gastric mucosa, causing gastritis in mice [41].

The PCR assay employed in this work specifically targets a region of the *ureC* (*glmM*) gene which has been shown to be unique and essential for the growth of *H. pylori*. It has been previously reported that detecting this gene improves sensitivity and specificity of recognition of *H. pylori* in samples containing prokaryotic cells as well as many organic impurities [9, 17, 37]. However, because the PCR assay detects *ureC* (*glmM*) gene of *H. pylori*, we are unable to speculate on the viability of organisms in water samples. 

The high prevalence of *H. pylori* isolated from healthy human carrier [42, 43] suggests that water contamination is due to poor hygiene management. Therefore, the consumption of tap water and dental units' water would be a potential risk of *H. pylori* infection for the consumer. To the author's knowledge, the present study is the first report of the isolation of *H. pylori* from water in Iran and the first demonstration of *H. pylori* DNA in tap water, dental units' water, refrigerated water with filtration, and public water cooler samples. Further studies will be necessary to determine the prevalence of *H. pylori* in water and other foods in Iran and to explore the potential risk of human infection with *H. pylori* via consumption of water and foods.

## Figures and Tables

**Table 1:** Frequency of *Helicobacter pylori* detected in different water samples in Iran by PCR.

Water sample	No. of samples	No. of *H. pylori*-positive by culture*	No. of *H. pylori*-positive by PCR
Tap water	50	2 (4.0%)	5 (10.0%)
Dental units' water	35	2 (5.8%)	4 (11.4%)
Bottled mineral water	20	0	0
Refrigerated water with filtration	30	0	1 (3.3%)
Tape water equipped with filtration system	25	0	0
Water cooler in public places	40	1 (2.5%)	4 (10.0%)

Total	200	5 (2.5 %)	14 (7.0%)

*Results expressed as the number of *H. pylori*-positive samples (percent positive samples analyzed).
